# Comparative Genomics of Prophages Sato and Sole Expands the Genetic Diversity Found in the Genus *Betatectivirus*

**DOI:** 10.3390/microorganisms9061335

**Published:** 2021-06-19

**Authors:** Annika Gillis, Louise Hock, Jacques Mahillon

**Affiliations:** Laboratory of Food and Environmental Microbiology, Earth and Life Institute, UCLouvain, Croix du Sud 2, L7.05.12, B-1348 Louvain-la-Neuve, Belgium; louise.hock@list.lu

**Keywords:** *Bacillus* phages, betatectiviruses, lysogeny, plasmidial prophages, tectivirus, temperate phages

## Abstract

Tectiviruses infecting the *Bacillus cereus* group represent part of the bacterial “plasmid repertoire” as they behave as linear plasmids during their lysogenic cycle. Several novel tectiviruses have been recently found infecting diverse strains belonging the *B. cereus* lineage. Here, we report and analyze the complete genome sequences of phages Sato and Sole. The linear dsDNA genome of Sato spans 14,852 bp with 32 coding DNA sequences (CDSs), whereas the one of Sole has 14,444 bp comprising 30 CDSs. Both phage genomes contain inverted terminal repeats and no tRNAs. Genomic comparisons and phylogenetic analyses placed these two phages within the genus *Betatectivirus* in the family *Tectiviridae*. Additional comparative genomic analyses indicated that the “gene regulation-genome replication” module of phages Sato and Sole is more diverse than previously observed among other fully sequenced betatectiviruses, displaying very low sequence similarities and containing some ORFans. Interestingly, the ssDNA binding protein encoded in this genomic module in phages Sato and Sole has very little amino acid similarity with those of reference betatectiviruses. Phylogenetic analyses showed that both Sato and Sole represent novel tectivirus species, thus we propose to include them as two novel species in the genus *Betatectivirus*.

## 1. Introduction

The *Bacillus cereus* group of Gram-positive bacteria, also known as *B. cereus sensu lato* (*s.l*), represents a very homogeneous cluster within the genus *Bacillus*, including more than 20 reported species that are genetically very close, but also display highly specialized lifestyles and virulence spectra [1,2,3]. Three members of the *B. cereus* group are very well studied, mainly due to their individual properties: *B. cereus sensu stricto (s.s.*) is a potential food contaminant and an opportunistic human pathogen, *Bacillus anthracis* is the causative agent of the lethal disease anthrax, and *Bacillus thuringiensis* is an entomopathogenic bacterium used worldwide as bioinsecticide [4]. However, the genomic situation of the *B. cereus* lineage is more complicated than a classification based on phenotypic properties and virulence spectra. In fact, the characteristics traditionally used to distinguish *B. anthracis*, *B. thuringiensis* and *B. cereus* are associated with multiple mobile genetic elements (MGEs) [4,5,6,7]. Therefore, it has been suggested that the *B. cereus* group have evolved from a common ancestor through genetic rearrangements involving the acquisition of mobile DNA such as plasmids, transposons, insertion sequences, and bacteriophages (phages for short).

Phages are recognized as shapers of bacterial genome architecture, having a very well-described impact on bacterial genetic diversity and behavior [8,9]. Members of the *B. cereus* group are known to be associated with many species-specific temperate (lysogenic) phages, either integrated into their chromosome or as independently replicating linear or circular elements (also known as plasmidial prophages) [5]. To date, temperate phages residing as linear plasmids in the *B. cereus* group have been identified as belonging to the genus *Betatectivirus* within the family *Tectiviridae* [4,10,11,12]. Betatectiviruses are also closely related to the cryptic linear plasmid pBClin15 of *B. cereus* type strain ATCC 14579 [13]. These lysogenic phages might play an important role in the life cycle and ecological traits of their *Bacillus* host, as they can enhance swarming motility and decrease biofilm and sporulation capacities [14].

Tectiviruses are icosahedral tail-less phages within the membrane-containing group of phages that infect both Gram-positive and Gram-negative bacteria. In these viruses, the membrane is enclosed beneath the protein capsid and is composed of approximately equal amounts of virus-encoded proteins and lipids derived from the host cell plasma membrane [10,15]. The icosahedral capsid of ~55–80 nm in diameter has flexible and elongated spikes extending from their vertices that act as receptor-recognition complexes akin to those of adenoviruses [15,16]. The 15–18 kb-long linear double stranded DNA (dsDNA) genome is coiled within the lipid membrane. To deliver the tectiviral genome upon recognition of its receptor, the membrane transforms into a tubular structure protruding from one of the 12 vertices of the capsid [17]. The genome contains inverted terminal repeats (ITRs) sequences and is capped with terminal proteins covalently linked at both 5′-ends, which facilitate the genome replication by means of a protein-primed mechanism resembling that of phage phi29 [18].

Currently, as officially approved by the International Committee on Taxonomy of Viruses (ICTV), the genus *Betatectivirus* includes four viral species all identified infecting members of the *B. cereus* group: *Bacillus virus* *Bam35* (type species), *Bacillus virus GIL16*, *Bacillus virus AP50* and *Bacillus virus Wip1* (hereinafter referred to as Bam35, GIL16, AP50 and Wip1, respectively) [19]. Phages Bam35 and GIL16 were isolated from *B. thuringiensis* strains, while AP50 and Wip1 were isolated from soil samples and infect specifically *B. anthracis* [5,10]. Other phage isolates like GIL01 [20] and GIL02 [21] are considered essentially the same phage as Bam35 because their genomes differ by few nucleotides [4,19]. Betatectiviruses share between 67 to 100% identity on DNA level. On average, their genomes are 14.7 kb in length, with 39.1 mol% G + C, and encode approximately 30 putative proteins and no tRNAs [19]. Apart from these six betatectiviruses that are fully sequenced, more than 50 other betatectiviruses infecting the *B. cereus* group have been uncovered and partially sequenced [10,22], along with several betatectiviruses-like molecules identified in other Gram-positive bacteria by databases mining [11]. Among these betatectiviruses, a highly variable region (HVR) [23] was shown to harbor unique and small genes with no orthologous (ORFans) in most otherwise well-conserved group of tectiviruses, suggesting that the acquisition of those ORFans may provide a source of genetic diversity within this group of phages [22]. In view of these findings, in this work we focused on two phages, Sato and Sole, by exploring their genomic and genetic diversity. The whole genome sequences of these phages were compared to those of other phages within the family *Tectiviridae*. Phylogenetic analysis and pairwise intergenomic distances between all accepted members of the family *Tectiviridae*, confirmed that phages Sato and Sole are two new species of tectiviruses and we propose to include them in the genus *Betatectivirus*.

## 2. Materials and Methods

### 2.1. Bacterial Strains, Phages and Culture Conditions

The following *B. cereus s.l.* strains were used in this study: cereulide-producing (emetic) *B. cereus* AND1284 and *B. cereus s.l.* strain VD166, which are the natural hosts of temperate phages Sato and Sole, respectively [22]; emetic *B. cereus* CER020 and *B. thuringiensis* GBJ002 that have been reported as sensitive hosts for phages Sato and Sole, respectively [10]; and *B. thuringiensis* GBJ002/GIL16, which is a lysogenic strain for reference tectivirus GIL16 [14]. All bacterial cultures were grown in lysogeny broth (LB) medium (5 g/L NaCl, 5 g/L yeast extract, 10 g/L tryptone). For agar plates (LB-agar), LB medium was solidified with 1.4% (wt/vol) agar. Overnight cultures were prepared by inoculating a single colony growing on LB-agar plates into 10 mL LB medium and incubating the cultures for 14 to 15 h at 30 °C and with agitation (120 rpm), unless otherwise stated.

### 2.2. Production of Concentrated Phage Stocks

Phage stocks were obtained by mitomycin C induction as previously described [10]. Briefly, 20 mL of a mid-log-phase culture of strains AND1284 and VD166 were treated with 100 ng/mL of mitomycin C (AppliChem) for 1 h. After washing the cultures twice with 0.01 M MgS0_4_, the cells were resuspended in 20 mL fresh LB medium and phage induction proceeded for 2 h at 30 °C and 120 rpm. Cultures were then centrifuged at 4500 rpm and 4 °C for 10 min. Supernatants were filtered (0.22-µm filter, Millipore) and phage stocks titers were determined by the double-layer agar method using phage Sato and Sole respective sensitive hosts (i.e., strains CER020 and GBJ002, respectively).

To obtain highly concentrated phage preparations, a polyethylene glycol (PEG) phage precipitation protocol was carried out as follows. Sodium chloride at a final concentration of 1.5 M and PEG 6000 (20%, wt/vol) were added to 10 mL of phage stocks and samples were stored at 4 °C for 24 h. Samples were then centrifuged at 15,000 rpm and 4 °C for 15 min and pellets were carefully resuspended in 1 M Tris Magnesium buffer (50 mM Tris-HCl, 10 mM MgS0_4_). Concentrated phage stocks titers were further assessed.

### 2.3. Phage DNA Extraction, Sequencing and Assembly

To 750 µL of concentrated phage stock, 3.75 µL of nuclease mix (0.25 mg/mL DNase I, 0.25 mg/mL RNase A, 50% glycerol, 150 mM NaCl) was added, gently mixed by inversion, and incubated at 37 °C for 10 min [24]. After a step of nuclease inactivation, phage DNA was obtained by phenol extraction and ethanol precipitation [25]. Purified phage DNA was resuspended in 30 µL of nuclease free water (Promega) and quantified by nanodrop (Thermo Scientific). Phage DNA integrity was verified by gel electrophoresis.

Phage DNA sequencing was performed at Macrogen Inc. (South Korea) using an Illumina MiSeq 250-bp paired-end platform with a 550-bp insert library (Truseq DNA PCR Free). High-throughput sequence data for both phages were analyzed and assembled as previously described [26]. Geneious Prime (v2020.2.2) was used to verify the assemblies, map the total reads on the assembled genome, and test for the presence of terminal repeats. The correct order between contigs was verified by gap-spanning and Sanger-sequencing (Macrogen Inc., The Netherlands) using primers listed in Table S1. Runoff sequencing was used to verify genome ends.

### 2.4. Phage Genome Annotation and Analysis

Phage genome annotation was initially assessed by Geneious Prime (v2020.2.2) by use of the “annotate from database” function that employs a BLASt-like algorithm to search for annotations that match the sequence and aligns it against the full length of each annotation. The database used for these annotations was constructed with the genomes of fully sequenced phages belonging to the genus *Betatectivirus* [19]. Annotation parameters comprised a minimum of 60% of sites covered that must be identical to transfer the annotation. Automated coding DNA sequences (CDSs) calls and annotation were independently assessed using RASTtk [27], FgenesV (http://www.softberry.com/; accessed on 16 April 2021), Glimmer (v3.02) [28] and GeneMarkS (v3.25) [29]. Further functional annotation was manually completed by BLASTp against the non-redundant protein database in NCBI with default parameters. (Pro)phage genome was also analyzed with PHASTER [30]. tRNAs were predicted using tRNAscan-SE (v2.0) [31] and membrane protein topology was predicted with the TMHMM server (v2.0) [32]. Phage genome maps were plotted using Geneious Prime (v2020.2.2) and postulated gene functions were manually added.

Nucleotide and amino acid similarities among betatectiviruses, including tectiviral-like plasmids, were analyzed and plotted with Easyfig (v2.1) using BLASTn and tBLASTx, respectively [33]. For pairwise analyses of *Betatectivirus* genomes and single stranded DNA (ssDNA) binding proteins (SSBs) similarities, sequences were aligned using MUSCLE [34] and computed by SDT (v1.2) [35]. To retrieve Sato and Sole SSBs homologous proteins, HHpred searchers [36] were done using SSBs from Sato and Sole as queries and the results manually inspected to select proteins with known OB-fold to be included in phylogenetic reconstructions (see below). SSBs secondary structure predictions were also carried out using Phyre2 [37] and I-TASSER [38] platforms. Multiple-amino acid-sequence alignments of terminal proteins were performed using CLUSTAL W [39]. In addition, phage genomes were subjected to a megaBLAST search against the NCBI nucleotide collection (nr/nt) database (analysis performed on March 31, 2021) to identify other closely related molecules (plasmids or (pro)phages). Any record having at least 90% identity and 85% query coverage was recovered.

### 2.5. Detection of a Plasmidial Prophage State by Plasmid Gel Electrophoresis

Plasmid profiles of *B. cereus s.l.* strains AND1284 and VD166 were prepared as described elsewhere [40] from overnight cultures grown in 7 mL of brain heart infusion (BHI) broth for 15 h at 30 °C and with agitation (120 rpm). *B. thuringiensis* GBJ002/GIL16 was used as a control to estimate the ~15 kb plasmidial prophages sizes. Plasmid bands corresponding to ~15 kb were excised from the agarose gel and further purified by use of a QIAquick gel extraction kit (QIAGEN). To confirm that the purified excised plasmid bands corresponded to tectiviruses Sato and Sole, PCRs amplifications were performed using Q5 polymerase (NEB) and primers pairs Sato-787-F and Sato-5500-R for phage Sato, and Sole-258-F and Sole-6500-R for phage Sole (Table S1). PCR amplicons were purified using a GenElute PCR cleanup kit (Sigma) and Sanger sequenced (Macrogen Inc., The Netherlands) using internal primers (Table S1). Obtained nucleotide sequences were trimmed and aligned against the genome sequences of Sato and Sole using Geneious Prime (v2020.2.2).

### 2.6. Phylogenetic Methods

Nucleotide and protein sequences for phages belonging to the family *Tectiviridae* were obtained from the NCBI GenBank and RefSeq databases. Multiple-amino acid-sequence alignments of the B-family DNA polymerase (DNAPolB), packaging ATPase and major capsid protein were obtained using MUSCLE [34] implemented in MEGA X [41]. The phylogeny of these canonical proteins was reconstructed in MEGA X by using maximum likelihood inference and the general reverse transcriptase model and tested by resampling of a value of 1000 bootstrap. Phylogenetic reconstructions between the SSBs of Sato and Sole and other SSBs, including other protein-primed replicating phages, were executed as those for canonical proteins. For alphatectiviruses and betatectiviruses, genome-wide nucleotide alignments were also generated with MUSCLE and phylogenetic analyses were conducted in MEGA X by maximum likelihood inference, using the general time-reversible plus gamma model of nucleotide substitution with a bootstrap value of 1000 iterations. Pairwise intergenomic similarity values between fully sequenced tectiviruses were calculated by the Virus Intergenomic Distance Calculator (VIRIDIC) tool [42]. As recommended by the ICTV, a >95% DNA sequence identity was used as species demarcation criterion and was calculated by BLASTn (% identity multiplied by % coverage) [43].

### 2.7. Nucleotide Sequence Accession Numbers

The annotated genomes of tectiviruses Sato (Bacillus phage Sato) and Sole (Bacillus phage Sole) were deposited in NCBI GenBank under the accession number of MZ089978 and MZ089979, respectively.

## 3. Results and Discussion

### 3.1. Genomic Features of Tectiviruses Sato and Sole

Phage Sato was initially discovered in the emetic *B. cereus* strain AND1284, whereas phage Sole was uncovered in strain VD166, a *B. cereus s.l.* strain isolated from soil, by screening for the presence of tectiviral prophage DNA and targeting the HVR present in reference betatectiviruses [22]. Further analysis of a region that comprised the HVR (~1000 bp) in Sato and Sole, along with other three tectiviruses discovered in the same study (i.e., phages Emet, Sand, and Lima) indicated that this region included some ORFans (i.e., open reading frames (ORFs) with no detectable homologs in sequence databases and thus no predicted function) located upstream and downstream a conserved regulatory core that includes a LexA repressor homologue [22,44]. To gain further insights into the genetic diversity displayed by tectiviruses Sato and Sole, DNA from both phages was extracted and sequenced on an Illumina MiSeq platform, combined with Sanger sequencing.

**Figure 1:**
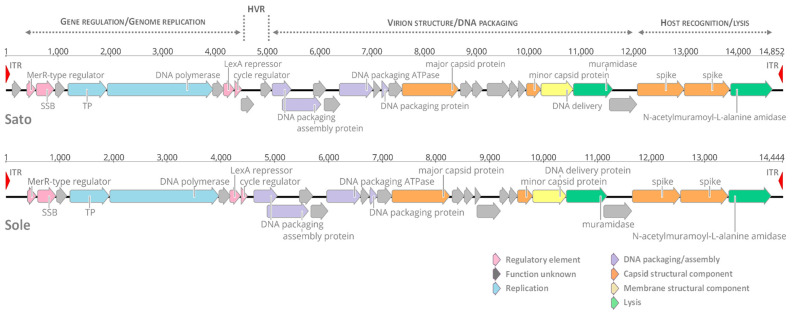
Genetic maps of tectiviruses Sato (**top**) and Sole (**bottom**). Predicted genes and their direction of transcription are represented as block arrows. Inverted terminal repeats (ITR) are shown as red arrows at both ends of the genomes. The color key at the bottom-right indicates known and postulated gene functions [4,10,22,23,44,45,46]. Three genetic modules based of functional grouping, together with the highly variable region (HVR), are indicated above the genomes. The rulers represent base pairs in the phage genomes. MerR-type, a putative transcriptional regulator; SSB, ssDNA binding protein; TP, terminal protein.

According to read analysis, assembly and annotation, phage Sato has a linear dsDNA genome of 14,852 bp in length, with 39.2 mol% G + C, and encodes 32 predicted CDSs (Figure 1, top). On the other hand, sequencing of phage Sole genome resulted in a linear 14,444-bp dsDNA molecule, with 39.6 mol% G + C, and having 30 predicted CDSs (Figure 1, bottom). None of these two phages present tRNAs in their genomes. Both phage genomes are terminated on each end by imperfect ITRs of 74 bp in length and encode for homologs of the canonical tectiviral proteins (DNAPolB, packaging ATPase and major capsid protein) and other well-conserved proteins among betatectiviruses (Figure 1).

### 3.2. Tectiviruses Sato and Sole Are Plasmidial Prophages

**Figure 2:**
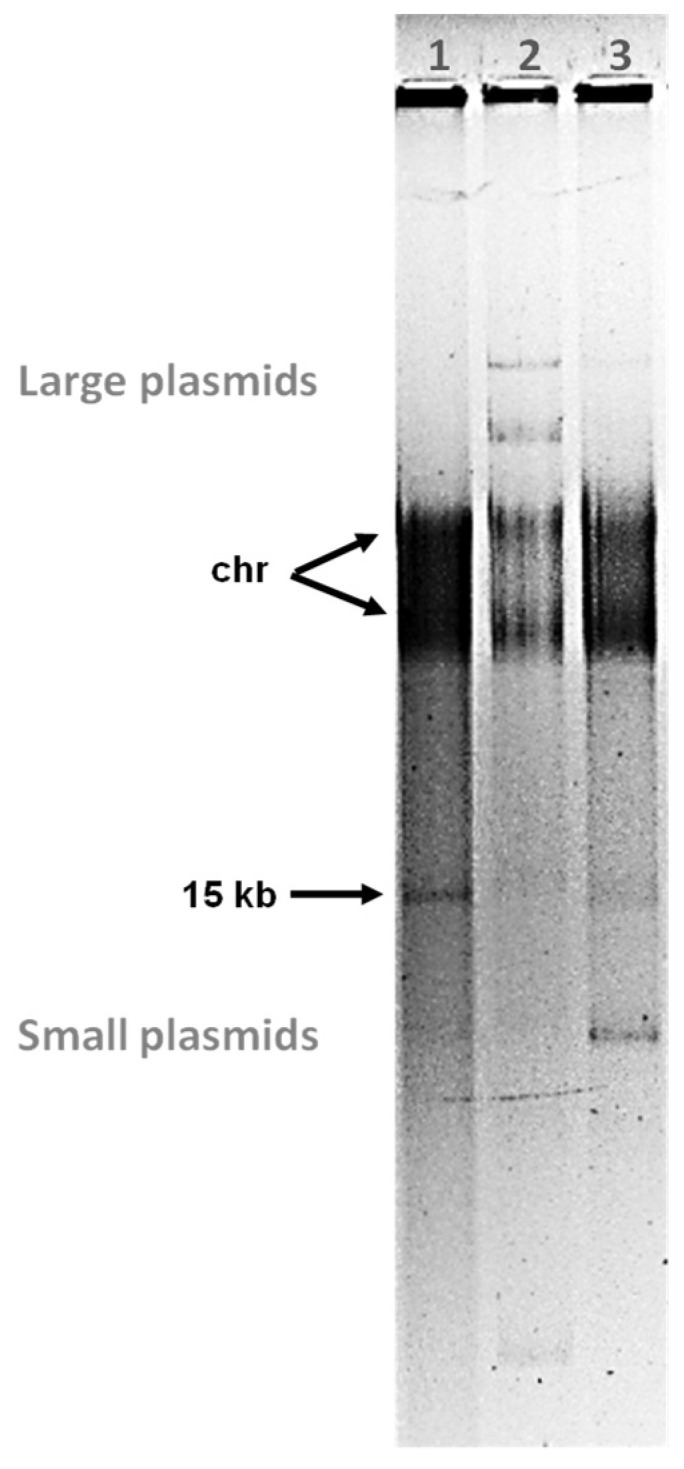
Plasmid(s) electrophoresis profiles of strains (1) *B. thuringiensis* GBJ002/GIL16; (2) emetic *B. cereus* AND1284; and (3) *B. cereus s.l.* VD166, harboring plasmidial tectiviruses GIL16, Sato and Sole, respectively. The tectiviral prophages in a linear plasmid state (~15 kb) are indicated. chr, linearized chromosomal DNA.

In previous studies, the temperate nature of tectiviruses Sato and Sole was established by phage induction via the DNA-damaging agent mitomycin C and producing stable lysogens in susceptible hosts [10,22]. To further confirm the plasmidial prophage state of Sato and Sole when infecting their respective *B. cereus* hosts, total bacterial plasmid extractions were prepared and electrophoresed in agarose gels. It is important to note that no phage induction treatment was applied prior to plasmid extractions in order to preserve the lysogenic state of the phages. Analysis of the plasmid content of *B. cereus* strains AND1284 and VD166 revealed the presence of a molecule of ~15 kb migrating like that of tectivirus GIL16 when in plasmidial prophage state (Figure 2). Both strains AND1284 and VD166 also harbor other large and small plasmids, as shown in Figure 2. Moreover, the ~15-kb band corresponding to phage Sole is migrating “faster” on the agarose gel than the bands that correspond to tectiviruses GIL16 and Sato. In fact, this observation fits with the determined genome length for these tectiviruses: Sole, 14,444 bp; GIL16, 14,844 bp; and Sato, 14,852 bp. The 15-kb plasmid bands from *B. cereus* strains AND1284 and VD166 were further excised from the agarose gel, purified, and verified by PCR and amplicon Sanger sequencing that they correspond to phages Sato and Sole, respectively (data not shown). These results confirm that both Sato and Sole can stablish a plasmidial prophage state in the host during their lysogenic cycle.

It is worth noting that taking advantage of the temperate nature of betatectiviruses, Jalasvuori and Koskinen [11] previously proposed that the supercont1.11 (GenBank acc. NZ_JH791864) from the draft genome of *B. cereus* VD166 [47] corresponds to the genome of phage Sole. The original annotation of supercont1.11 contained 22 putative CDSs. In the present study, we confirmed by phage induction, phage DNA extraction and whole genome sequencing, that the genome of Sole corresponds to that of *B. cereus* VD166 supercont1.11. Nevertheless, a single nucleotide deletion was pinpointed in the genome assembled in this work (i.e., 14,444 bp in length vs. 14,445 bp of that of supercont1.11). This deletion was detected in position 4514 of Sole genome where a T is missing in a stretch of other eight Ts located in the intergenic region that corresponds to the HVR. To confirm that this deletion did not represent a sequencing or assembly error, PCRs performed on Sole phage DNA preparations and on the ~15-kb plasmid band extracted from agarose gels were subjected to amplicon Sanger sequencing. The results confirmed that the genome of phage Sole is 14,444 bp in length and the CDS assignment and annotation refinement performed in this work identified 30 putative encoded proteins (Figure 1).

### 3.3. Comparative Genomics of Tectiviruses Sato and Sole

**Figure 3:**
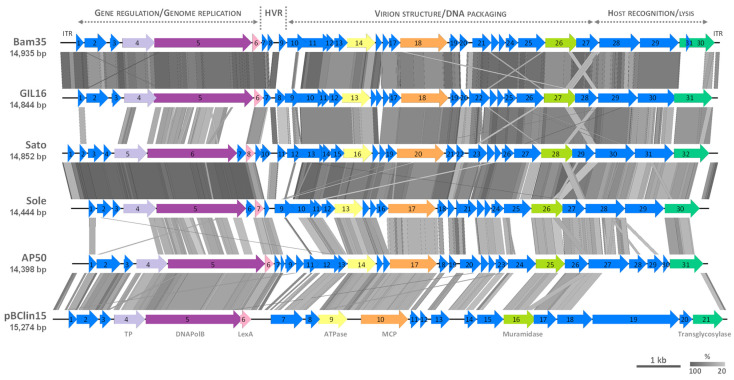
Genome comparisons of phages Sato and Sole with other betatectiviruses and the tectivirus-like element pBClin15. Predicted genes and direction of transcription are represented as block arrows. CDSs numbers are indicated inside the block arrows. Canonical tectiviral proteins and well conserved proteins among betatectiviruses are color-coded: light purple, terminal protein (TP); dark purple, B-family DNA polymerase (DNAPolB); rose, LexA transcriptional regulator; yellow, packaging ATPase (ATPase); orange, major capsid protein (MCP); light green, muramidase; dark green, transglycosylase. The other CDSs are in blue. ITR, inverted terminal repeat; HVR, highly variable region. Conserved regions are grey-shaded, with the color intensity indicating the percentage of amino acid identity. The comparisons were done by tBLASTx, and similarities with E values lower than 0.001 were plotted using EasyFig (v.2.1) [33]. Scale and percentage of amino acid identity are indicated at the bottom-right. Genome lengths in base pairs (bp) are indicated for each molecule. GenBank accession numbers are listed in Table 3.

As for other betatectiviruses, Sato and Sole display a modular genome that can be divided into three functional modules, besides the HVR: (*i*) gene regulation-genome replication, (*ii*) virion structure-DNA packaging, and (*iii*) host recognition-lysis (Figure 1) [10,23]. The first module is also referred to as the “plasmid region”, because it encodes proteins that ensure genome replication as a linear plasmid inside the host cell and lysogeny control. The other two modules are contained within the “phage region” which encodes all the virion structural and DNA packaging proteins, together with the lytic enzymes [5,10]. In the case of phage Sato, its “plasmid region” contains two putative proteins (encoded by CDSs 1 and 7) of unknown function, which are not present in the reference tectiviruses Bam35, GIL16 and AP50 (Table 1 and Figure 3). Wip1 genome was not included in the analyses as the genes governing gene regulation-genome replication are scattered along its genome and, therefore, there is not a clear delimited “plasmid region”. Sato CDS 7 is also present in phage Sole genome (CDS 6), sharing 99.54 and 100% identity at the nucleotide and amino acid level, respectively. On the other hand, no orthologous gene for Sato CDS 1 was detected in phage Sole genome (Table 1) and in the databases, thus the transcription and translation of this ORFan into a protein remains to be demonstrated, along with the CDSs 6 and 7 in phages Sole and Sato, respectively.

Previously it was suggested that the “plasmid region” of phages Sato and Sole harbors a greater genetic diversity than previously observed among that of reference betatectiviruses [10]. In fact, while studying two variable regions, one located in the “plasmid region” and other in the “phage region”, phages Sato and Sole were not detected by probes that amplified the former region, specifically the C-terminal end of the DNAPolB gene (CDS 5 in Bam35, Table 1), albeit they were detected with probes that targeted the latter region [10]. In this work, genomic comparisons performed with BLASTn and tBLASTx of the three functional modules of phages Sato and Sole with that of reference betatectiviruses, including the tectiviral-like plasmid pBClin15, revealed a low sequence similarity with other tectiviruses in the “gene regulation-genome replication” module (plasmid region) of Sato and Sole (Figure 3 and Figure S1), despite of harboring genes coding for a DNAPolB (canonical tectiviral protein), a putative SSB, and a putative terminal protein (Table 1 and Table 2). Conversely, the two functional modules that constitute the “phage region” of tectiviruses Sole and Sato displayed both nucleotide and amino acid sequence homologies with that of reference tectiviruses (Figure 3 and Figure S1). Nevertheless, in the “phage region” of Sato and Sole the module of “host recognition-lysis” showed a lesser similarity conservation than the “virion structure-DNA packaging” module when compared to other tectiviruses and among Sato and Sole themselves.

Further protein homology searches of common proteins encoded in the “plasmid region” of reference betatectiviruses Bam35, GIL16 and AP50 (Table 2) revealed that the LexA-type repressor protein (CDS 6 in Bam35) shared the highest percentage of amino acid identity (60.6–77.3%) when compared to those of phages Sato and Sole, followed by the MerR-type regulator (CDS 1 in Bam35) (36.7–40.4%) and the DNAPolB (CDS 5 in Bam35) (38.8–39.6%). The protein of unknown function, encoded by CDS 3 in Sole and CDS 4 in Sato, only displayed 25 and 33.3% of amino acid identity, respectively, with that of tectivirus GIL16, suggesting that this CDS is likely to be a source of genetic diversity not only for the phage itself, but also for their bacterial hosts as it is encoded by the “plasmid region”. The actual function of this protein in all betatectiviruses remains to be elucidated.

While some degree of similarity was detected among the proteins previously mentioned, BLASTp analyses (Table 2) strikingly did not show any percentage of amino acid sequence identity between the SSB proteins of phages Sato (CDS 3) and Sole (CDS 2) and those of Bam35, GIL16 and AP50, despite that protein structure prediction and modeling [36,37,38] identified them as SSBs, with the best hits being homologous proteins found in *Staphylococcus aureus* (PDB 5XGT) (Figure S2a), *Helicobacter pylori* (PDB 2VW9) and *Bacillus subtilis* (PDB 3VDY). Further additional pairwise amino acid sequence analysis [35] of Sato and Sole SSBs indicated that they shared between 14 to 22% sequence identities with those of Bam35, GIL16 and AP50 (Figure S3). In general, SSBs are essential for DNA replication. These proteins are found in all domains of life and in many viruses, but the proteins themselves are very diverse with little sequence similarity, subunit composition, and oligomerization states. Most SSBs contain at least one OB-fold (DNA-binding oligonucleotide/oligosaccharide binding) domain [52,53]. Recently it was shown that Bam35 SSB binds ssDNA in a highly cooperative, yet sequence-independent manner. Additionally, Bam35 SSB and the phage phi29-related SSB proteins, seem to be forming a novel clade of SSBs from protein-primed replicating viruses that share a highly divergent OB-fold-like domain [46]. Maximum likelihood phylogeny reconstruction among SSB proteins with OB-fold (Figure S2b) indicated two well separated clusters: one grouping betatectiviruses Sato and Sole with more classical OB-fold SSBs, including the previously mentioned SSBs from *S. aureus, B. subtilis* and *H. pylori* and, the second cluster containing other betatectiviruses (i.e., Bam35, GIL01, GIL16 and AP50) along with protein-primed replicating phages belonging to the subfamily *Picovirinae* (family *Salasmaviridae*). Further functional studies on the SSBs encoded by phages Sato and Sole, in combination with phylogenetic and structural analyses, are required to understand their role in the phage DNA replication mechanism.

The replication of tectiviral genomes follows the basic principle of the well-known protein-primed mechanism from phage phi29 (reviewed in [54,55]). In this mechanism the terminal protein is 5′-covalently linked to the genome ends and act as a primer in the initiation of the replication process. For betatectivirus Bam35 is has been shown that the terminal protein is bound to the genome ends through a phosphodiester bond between the beta-hydroxyl group of tyrosine 194 (Y194) and the 5’-phosphate of the terminal deoxythymidylate. Thus, Y194 represents the priming residue. Additionally, tyrosine 172 (Y172) plays a key role in the interaction with the DNA polymerase [18]. Multiple sequence alignments between terminal proteins of betatectiviruses showed that these two key tyrosine residues are also conserved in the terminal proteins of phage Sato and Sole (Figure S4), suggesting that they fulfill similar functions as for Bam35. Furthermore, the ITRs sequences at both genome ends contain the DNA replication origins. In the case of phage phi29, the genome ends of the template strand are ^3′^TTTCAT…^5′^ and replication initiation occurs at the second nucleotide of this terminal repetition. To recover the full-length genome, a so called “sliding-back mechanism” occurs, where the terminal protein-dAMP initiation complex translocates backwards one position to recover the template information corresponding to the first ^3′^T, which in turn will act as template to incorporate the next nucleotide [18,55]. In alphatectivirus PRD1 replication starts at the fourth nucleotide of the terminal repetition ^3′^CCCCTAT…^5′^ and uses a “stepwise sliding-back mechanism” to recover the three last nucleotides [56]. In contrast, betatectivirus Bam35 replication initiation is mediated by the third nucleotide in the terminal repetition ^3′^ATAATACC…^5′^ and the DNA information of the first two nucleotides is recovered by a “single nucleotide jumpling-back mechanism” [18]. Considering the genome ends of phages Sato (^3′^AAAACC…^5′^) and Sole (^3′^AAAAACC…^5′^) (Figure S5a) it is tempting to hypothesize that the terminal DNA information is recovered via a sliding-back mechanism like that of phage phi29, or alternatively as alphatectivirus PRD1, by means of the stepwise sliding-back mechanism (Figure S5b), rather than employing a jumping back process akin to betatectivirus Bam35. Future experiments that focus on the early replication steps of tectiviruses Sato and Sole will shed light not only on the role of the genome ends patterns and the type of mechanism used by these two phages to recover the full-length genome information, but also on the entailment of terminal protein residues “Y172” and “Y194” in the protein-primed DNA replication process.

### 3.4. Phylogenetic Position of Sato and Sole within the Family Tectiviridae

**Figure 4:**
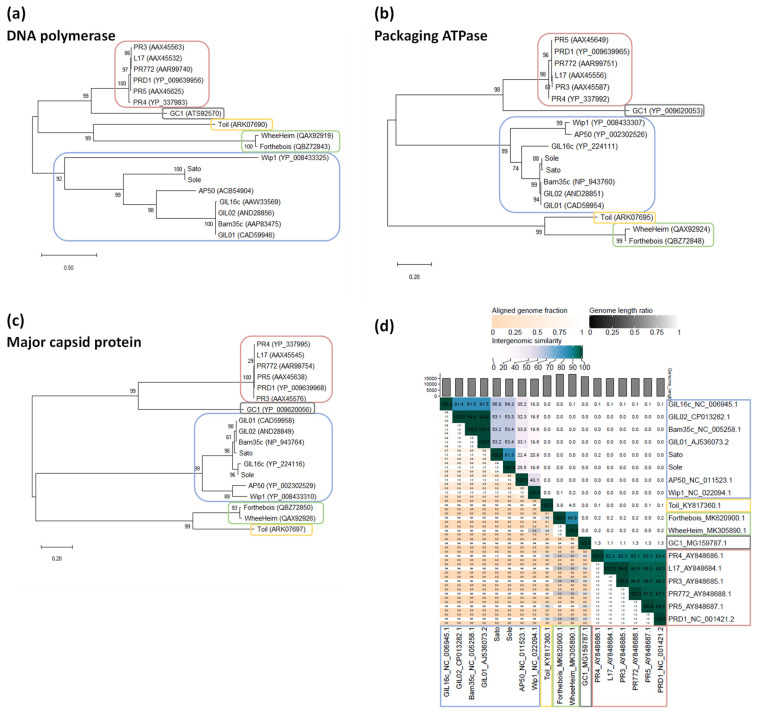
Phylogenetic relationships of phages Sato and Sole with other phages within the family *Tectiviridae*. (**a**–**c**) Phylogenetic trees resulting from maximum likelihood inference on multiple amino acid sequence alignments of the three canonical tectiviral proteins: the DNA polymerase (**a**), packaging ATPase (**b**), and major capsid protein (**c**). The general reverse transcriptase model was used to compute the maximum likelihood trees. Bootstrap values (1000 iterations) above 60% are indicated for each node. Scale bars in (**a**–**c**) represent the numbers of substitutions per site. Protein GenBank accession numbers are indicated in parentheses. (**d**) Virus Intergenomic Distance Calculator (VIRIDIC) [42] generated heatmap incorporating intergenomic similarity values (right half) and alignment indicators (left half and top annotation). Phage genome GenBank accession numbers are indicated. Color boxes (**a**–**d**) highlight phages belonging to genera *Alphatectivirus* (rose), *Betatectivirus* (blue), *Gammatectivirus* (grey), *Deltatectivirus* (green) and *Epsilontectivirus* (yellow). Note that Forthebois, WheeHeim and Toil are actinophages, isolated from the Gram-positive bacteria *Streptomyces scabiei* for the two formers [24] and from *Rhodococcus opacus* for the latter [58].

The taxonomical placement of phages Sato and Sole was further investigated taken into account genome organization and sequence identity comparisons (Figure 3 and Figure S1), in combination with phylogenetic analyses (Figure 4), the latter ones including phages belonging to the five currently accepted genera within the family *Tectiviridae*: *Alphatectivirus, Betatectivirus, Gammatectivirus*, *Deltatectivirus,* and *Epsilontectivirus* [12,24,57,58]. Maximum likelihood assessments with aligned sequences of the three canonical tectiviral proteins (i.e., DNAPolB, packaging ATPase and major capsid protein) showed that Sato and Sole clustered together with phages belonging to the genus *Betatectivirus* (Figure 4a–c). In addition, pairwise intergenomic distances analysis between completely sequenced tectiviruses, confirmed that Sato and Sole are members of the family *Tectiviridae* and validated their placement in the genus *Betatectivirus* (Figure 4d). Maximum likelihood analysis of complete genome sequences of alphatectiviruses and betatectiviruses, along with pairwise comparisons of nucleotide identity among fully sequenced betatectiviruses, also indicated that Sato and Sole formed a cohesive cluster within the genus *Betatectivirus* (Figure S6). Moreover, pairwise comparisons of phages Sato and Sole with other betatectiviruses using BLASTn, as recommended by the ICTV, showed that Sato and Sole shared between 17–56% nucleotide sequence identities with other betatectiviruses (Table 3). Based on the ICTV species demarcation criterion, we propose to include phages Sato (*Betatectivirus sato*) and Sole (*Betatectivirus sole*) as two new species within the genus *Betatectivirus*, as they share less than 95% DNA sequence identity between them (Table 3).

Finally, a megaBLAST search using Sato and Sole genomes as queries against the NCBI nucleotide collection was performed to identify other possible closely related phages. Genomes of two plasmids, pEFR-1-4 and pEFR-4-5, belonging to the *B. cereus* group were retrieved. Pairwise comparisons using BLASTn indicated that these plasmids shared 89 and 99% nucleotide identities with Sole and Sato, respectively (Table 3). Further genome organization and sequence identity comparisons (Figure S7) clearly suggested that plasmids pEFR-1-4 and pEFR-4-5 represent two putative Sato isolates that were sequenced as plasmids while sequencing the genome of their *Bacillus* host. Yet, phage induction, virion assembly and host lysis by these tectiviral-like molecules remain to be investigated.

**Table 3:** Percentage of nucleotide sequence identities between tectiviruses Sato and Sole against fully sequenced phages belonging to the genus *Betatectivirus* and tectiviral-related plasmids.

				Sato			Sole		
	Original Host	GenBank Acc. No.	Genome Length (bp)	Percent Cover	Percent Identity	% nt SequenceIdentity ^1^	Percent Cover	Percent Identity	% nt SequenceIdentity ^1^
Phage									
Bam35 ^2^	*B. thuringiensis*	NC_005258	14,935	60	85.82	51.49	55	88.96	48.93
GIL01	*B. thuringiensis*	AJ536073	14,931	60	85.84	51.50	60	88.60	53.16
GIL02	*B. thuringiensis*	CP013282	14,961	60	85.84	51.50	60	88.60	53.16
GIL16 ^2^	*B. thuringiensis*	NC_006945	14,844	66	84.43	55.72	59	92.52	54.59
AP50 ^2^	*B. anthracis*	NC_011523	14,398	29	66.38	19.25	31	65.81	20.40
Wip1	*B. anthracis*	NC_022094	14,319	28	65.01	18.20	26	66.70	17.34
Sato	*B. cereus*^3^	MZ089978	14,852	-	-	-	93	96.77	90.00
Sole	*B. cereus s.l.*	MZ089979	14,444	90	96.77	87.09	-	-	-
Plasmid									
pBClin15	*B. cereus*	AE016878	15,274	37	75.30	27.86	40	72.46	28.98
pEFR-1-4	*B. cereus*	CP064076	14,727	99	99.99	98.99	92	96.94	89.18
pEFR-4-5	*B. paranthracis*	CP064084	14,728	99	99.99	98.99	92	96.94	89.18

^1^ Determined by BLASTn (% identity multiplied by % coverage) as recommended by the ICTV [43]. ^2^ Clear plaque mutants were used for genome sequencing [13,23,59]. ^3^ Cereulide-producing (emetic) strain of *B. cereus* [22].

## 4. Conclusions

Phages are an important repository of genetic diversity and source of unexplored genes. In the *B. cereus* lineage of bacteria, the contribution of phages to its evolution and diversity has been largely overlooked. This work describes the genomic characterization of two tectiviruses, Sato and Sole, infecting the *B. cereus* group. We showed that these phages, like other tectiviruses infecting the *B. cereus* group (betatectiviruses), behave as linear plasmids during their lysogenic cycle. However, the mechanism by which these plasmidial prophages switch to their lytic cycle is not completely understood. In future experiments it would be most interesting to address if Sato and Sole lysogeny might have an impact in the life traits of their hosts, for example in the production of the emetic toxin cereulide. The genomic data analysis in this work also showed that phages Sato and Sole display the typical three genomic modules of those of reference betatectiviruses, having a conserved gene synteny on the whole genome. Still, the “plasmid region” of these two phages, that comprises the gene regulation-genome replication module, displays very low nucleotide and amino acid sequence similarity with other betatectiviruses. Based on phylogenetic analyses phages Sato and Sole can be tentatively placed within the genus *Betatectivirus* as two novel species. Overall, genome sequencing and comparative analysis of tectiviruses Sole and Sato have expanded the view of the genomic diversity occurring in plasmidial prophages found in members of the *B. cereus* group.

## Figures and Tables

**Table 1:** Comparison of CDSs encoded in the gene regulation-genome replication module of Sato and Sole to those of other reference betatectiviruses.

Phage CDSs (No. of Amino Acids Encoded)					Protein Function	Reference
Sato	Sole	Bam35	GIL16	AP50		
1 (54)	-	-	-	-	Hypothetical protein	This work
2 (56)	1 (56)	1 (58)	1 (58)	1 (61)	MerR-type regulator	[44]
3 (112)	2 (112)	2 (167)	2 (166)	2 (180)	ssDNA binding protein	[46,48]
4 (61)	3 (61)	3 (74)	3 (74)	3 (74)	Hypothetical protein	[4,13,45]
5 (245)	4 (245)	4 (245)	4 (245)	4 (233)	Terminal protein	[18,45]
6 (674)	5 (674)	5 (735)	5 (753)	5 (731)	DNA polymerase	[45,49,50]
7 (72)	6 (72)	-	-	-	Hypothetical protein	This work
8 (66)	7 (66)	6 (66)	6 (66)	6 (67)	LexA-type repressor	[44,51]

-, CDS not present in the phage genome.

**Table 2:** Percentage of amino acid sequence identities of common proteins encoded in the gene regulation-genome replication module of Sato and Sole and other betatectiviruses ^1^.

Protein	Sato vs. Sole	Sato vs.			Sole vs.		
		Bam35	GIL16	AP50	Bam35	GIL16	AP50
MerR-type regulator	100 (100)	36.73 (87)	36.73 (87)	40.43 (83)	36.73 (87)	36.73 (87)	40.43 (83)
ssDNA binding protein (SSB)	97.32 (100)	ND ^2^	ND	ND	ND	ND	ND
Unknown	95.08 (100)	ND	33.33 (39)	ND	ND	25.00 (39)	ND
Terminal protein	99.18 (100)	36.13 (93)	37.07 (91)	34.43 (37)	36.13 (93)	37.07 (91)	34.43 (37)
DNA polymerase (DNAPolB)	99.70 (100)	39.02 (93)	39.57 (93)	38.77 (93)	38.86 (93)	39.42 (93)	38.92 (93)
LexA-type repressor	78.46 (98)	77.27 (100)	77.27 (100)	63.08 (98)	63.08 (98)	63.08 (98)	60.61 (100)

^1^ Calculated by BLASTp. Results are displayed as percentage of identity followed by percentage of coverage in parentheses. ^2^ ND, no protein similarity detected.

## Data Availability

The data presented in this study is openly available in the GenBank repository.

## Supplementary Materials

The following are available online at https://www.mdpi.com/article/10.3390/microorganisms9061335/s1, Figure S1: Genome comparisons of phages Sato and Sole with other betatectiviruses and the tectivirus-like element pBClin15. Figure S2: (a) Structural models of Sato and Sole ssDNA binding proteins (SSBs) generated by Phyre2 server using *Staphylococcus aureus* SSB structure (PDB 5XGT) as template. (b) Phylogenetic relationships of SSBs. Figure S3: Graphical representation of percent pairwise amino acid sequence identity (in white) of the ssDNA binding protein in betatectiviruses. Figure S4: Multiple sequence alignment of betatectiviruses terminal proteins (TPs). Figure S5: (a) Genome ends sequences of betatectiviruses Sato and Sole and (b) schematic representation of the possible early steps involved in the protein-primed replication of these phages. Figure S6: Phylogenetic relationships of phages Sato and Sole with other alphatectiviruses and betatectiviruses. Figure S7: Genome comparisons of phages Sato and Sole with *B. cereus s.l.* plasmids pEFR-1-4 and pEFR-4-5. Table S1: Primers used in this study.
