# Supplementary material for: Comparative Genomics of Prophages Sato and Sole Expands the Genetic Diversity Found in the Genus Betatectivirus
Source: Microorganisms. 2021 Jun 19;9(6):1335. doi: 10.3390/microorganisms9061335 (PMC8234876; doi:10.3390/microorganisms9061335)
Supplement: Supplementary file 1 [file microorganisms-09-01335-s001.zip › microorganisms-1233108-supplementary.pdf]

## Supplementary Material

# Comparative Genomics of Prophages Sato and Sole Expands the Genetic Diversity found in the Genus *Betatectivirus*

Annika Gillis *, Louise Hock and Jacques Mahillon *

Laboratory of Food and Environmental Microbiology, Earth and Life Institute, UCLouvain, Croix du Sud 2, L7.05.12, B-1348 Louvain-la-Neuve, Belgium

* Corresponding authors:

Mailing address: Croix du Sud, 2-L7.05.12, B-1348 Louvain-la-Neuve, Belgium.
E-mail: annika.gillis@uclouvain.be (A.G.); jacques.mahillon@uclouvain.be (J.M.)

**Supplementary Material:**
- **Table S1**
- **Figures S1-S7**

**Table S1.** Primers used in this study.

| Phage Sato | | Phage Sole | |
|---|---|---|---|
| **Primer Name** | **Sequence (5′ → 3′)** | **Primer Name** | **Sequence (5′ → 3′)** |
| Sato-1-F | TTTTGGTTACCTGTGTCTATAATTCTT | Sole-1-F | TTTTTGGTTACCTGTGTCTATAATTCT |
| Sato-787-F | GGTCATCGTGTCGGAGTCAG | Sole-258-F | ACGCCCGTGTGTTCGTATAA |
| Sato-974-F | ACTCGAAAATGGAGAGCGCA | Sole-678-R | AACGTAAAAGTTGCCACCGC |
| Sato-995-R | AGTGCGCTCTCCATTTTCGA | Sole-1363-F | GCAGAAGCAAAAAGGTGGGG |
| Sato-2844-R | CCCGTTTTTCCCACCGGATA | Sole-2978-F | ATGGCGGAATCATTTGGGGT |
| Sato-3159-R | TCTCATAGCAAAACGCCCGT | Sole-2997-R | ACCCCAAATGATTCCGCCAT |
| Sato-5140-F | CTTTCGCTTGCGATTCGGAG | 4392F * | GGGTATATGGAAGAGTTTGG |
| Sato-5223-R | TCGTCATCGTCGATGTCGTC | Sole-4846-F | AGGAGGATGCACCACAATGG |
| Sato-5500-R | TCTTTTCCTCTTCAGACAGTTCC | 4940R * | CTCCTTATCGAATGTTTG TTC |
| Sato-6270-F | GCGTTATTACCGCGTGTTCC | Sole-6481-F | TGACCGTGACCGCATTGTAA |
| Sato-7010-R | AGTTTCAAAGTGGCGCGAAC | Sole-6500-R | TTACAATGCGGTCACGGTCA |
| Sato-9100-F | GAAGCGGGGAGAAGAAATGAGT | Sole-6600-R | AGTTTCAAAGTGGCGCGAAC |
| Sato-9312-F | TGATGACGCCGGACTTATCG | Sole-6986-F | ACGTTTGCGCAACTTGTTGA |
| Sato-9331-R | CGATAAGTCCGGCGTCATCA | Sole-7056-R | AACACCTCGTCCGATGTGAC |
| Sato-9427-R | TTCTTTTTGCGCGTCCCTTG | Sole-9120-F | TAAGGGGTGGCTCGGATACA |
| Sato-11234-F | CGAATTATGCGGGGCAACTG | Sole-9139-R | TGTATCCGAGCCACCCCTTA |
| Sato-11253-R | CAGTTGCCCCGCATAATTCG | Sole-9817-F | AAACTCGACCCTCCTGCAAG |
| Sato-13392-R | ATCGGCGGGGAATCTTGTTT | Sole-9859-R | TATGTTTTCCACCACCCCCG |
| Sato-13708-F | TTACAGTAAGGCAAGCGGGG | Sole-10389-R | ATTTGGCGCGTCTTTTGCTT |
| Sato-14000-R | GTTAATTCCGTTCTCTTCGCAT | Sole-10873-F | AACGCCATACACCGGAGAAG |
| Sato-14491-R | ACACGCTTTTTAACACCCGC | Sole-11784-F | GTTCGTGTCCTACCGAGTCC |
| | | Sole-13807-R | CTCCACGGTTTGCGATACCT |
| | | Sole-14138-R | GATTGCCATTCGCTACCACG |
| | | Sole-14151-R | CGTACTACCCCATGATTGCCA |
| Sato-Ends-390R | CACCTACTTTCAGTTCATTTCGGT | Sole-Ends-388R | CCTACTTTCAGTTCATTTCGGTTGT |
| Sato-Ends-14570F | CGGAAGTAAAGGACAAGCGC | Sole-Ends-14154F | TGGAGAAGGTGGATATACATGGT |

* From ref. [1].

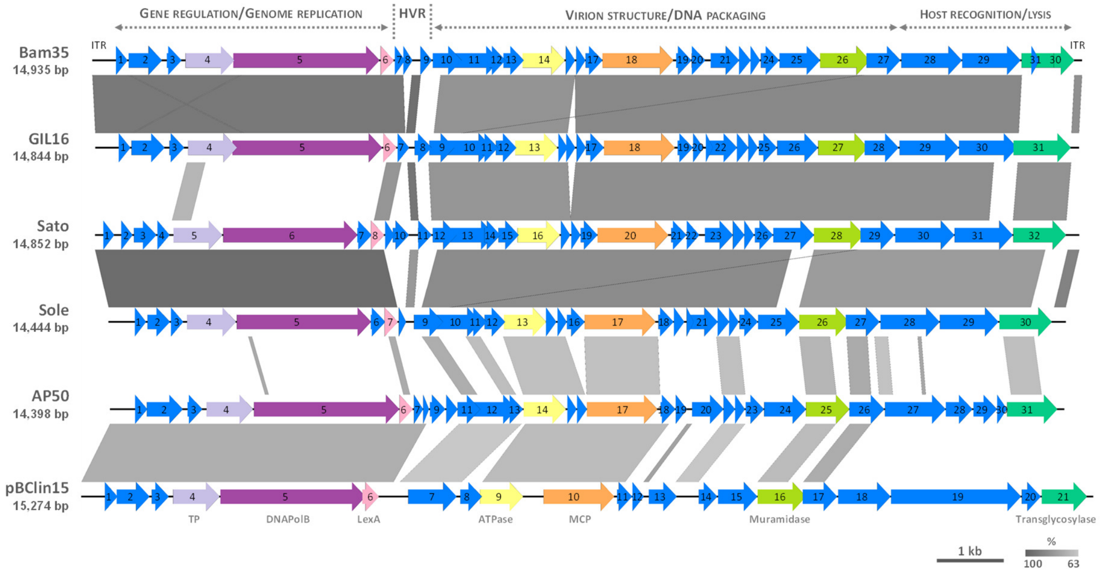


**Figure S1.** Genome comparisons of phages Sato and Sole with other betatectiviruses and the tectivirus-like element pBClin15. Predicted genes and direction of transcription are represented as block arrows. CDSs numbers are indicated inside the block arrows. Canonical tectiviral proteins and well conserved proteins among betatectiviruses are color-coded: light purple, terminal protein (TP); dark purple, B-family DNA polymerase (DNAPolB); rose, LexA transcriptional regulator; yellow, packaging ATPase (ATPase); orange, major capsid protein (MCP); light green, muramidase; dark green, transglycosylase. The other CDSs are in blue. ITR, inverted terminal repeat; HVR, highly variable region. Conserved regions are grey-shaded, with the color intensity indicating percentage of nucleotide identity. The comparisons were done by BLASTn, and similarities with E values lower than 0.001 were plotted. Scale and percentage of nucleotide identity are indicated at the bottom-right. Genome lengths in base pairs (bp) are indicated for each molecule. GenBank accession numbers are listed in Table 3.

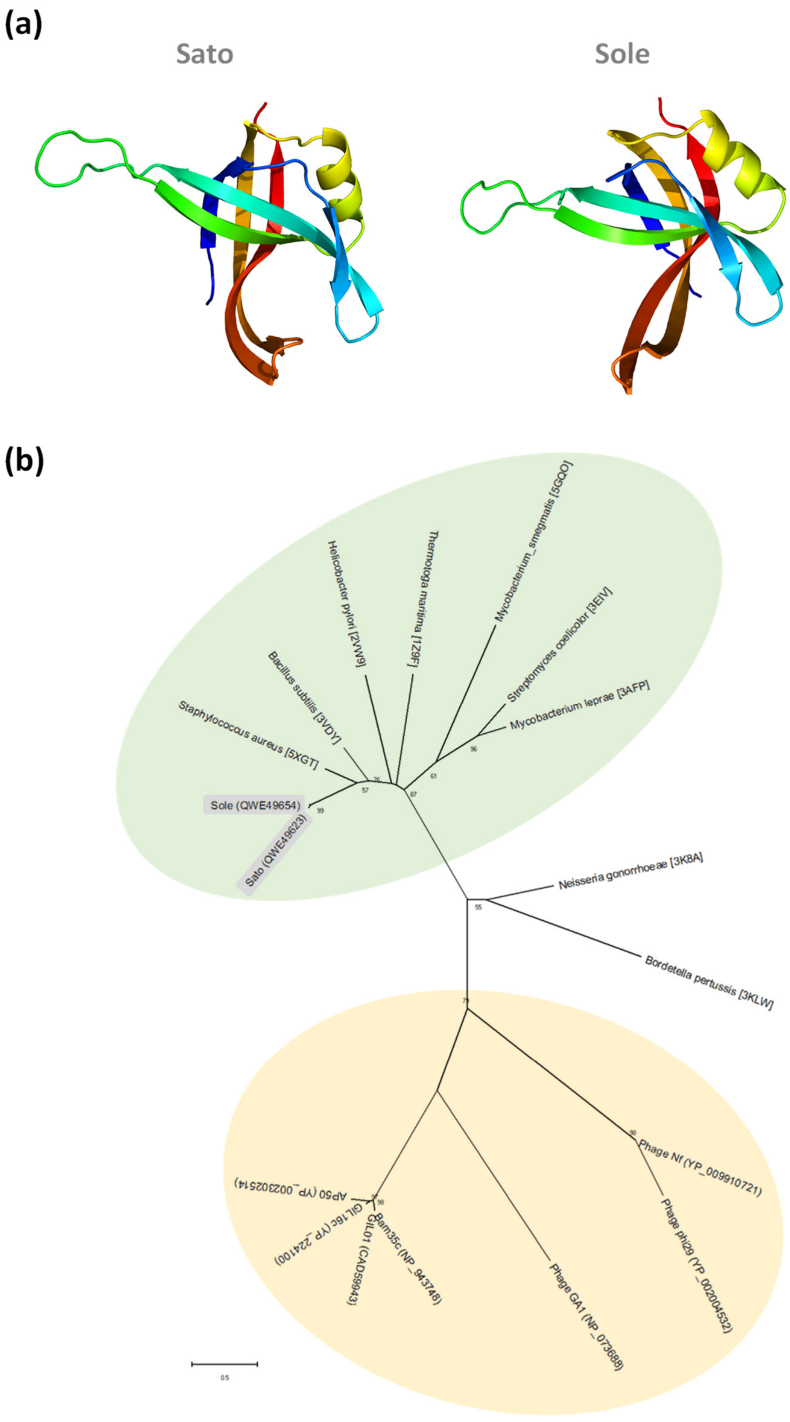


**Figure S2. (a)** Structural models of Sato and Sole ssDNA binding proteins (SSBs) generated by Phyre2 server [2] using *Staphylococcus aureus* SSB structure (PDB 5XGT) as template. **(b)** Phylogenetic relationships of SSBs. The general reverse transcriptase model was used to compute the maximum likelihood tree. Bootstrap values (1,000 iterations) above 55% are indicated for each node. Coloured areas highlight grouping of Sato and Sole SSBs (depicted in grey) with classical OB-fold SSBs (green) compared to a more divergent clade of SSBs formed by other betatectiviruses and protein-primed replicating *Picovirinae* phages (yellow). Primosomal replication proteins with OB-fold from *Bordetella pertussis* and *Neisseria gonorrhoeae* were also included in the analysis based on HHpred results (see Materials and Methods). Scale bar represents the numbers of substitutions per site. Protein GenBank or PDB accession numbers are indicated in parentheses or brackets, respectively.

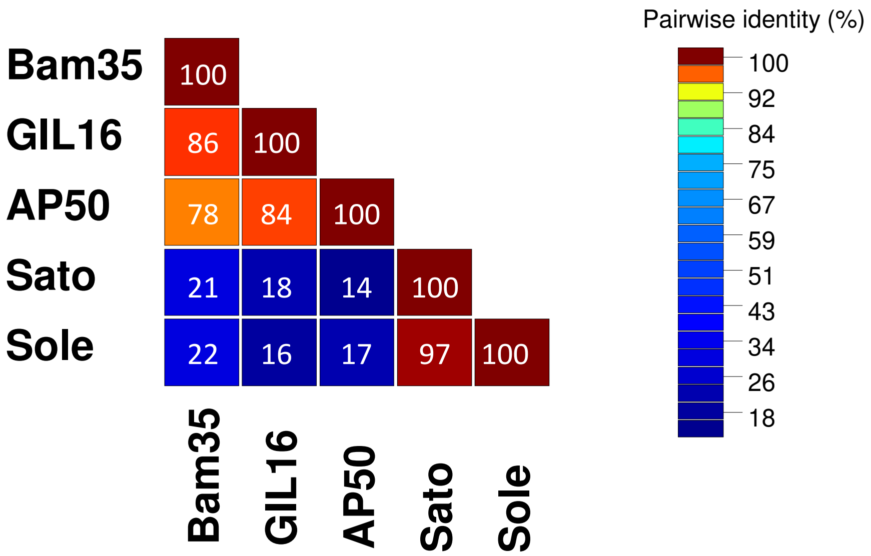


**Figure S3.** Graphical representation of percent pairwise amino acid sequence identity (in white) of the ssDNA binding protein in betatectiviruses. The color scale bar indicates the percentage of amino acid identity.

```
              ....|....| ....|....| ....|....| ....|....| ....|....| ....|....| ....|....|
                     10         20         30         40         50         60         70
Bam35c        MANKRLKKKL ETKRKKSLLV SEG-YSKKET KKLKGRELET VYKKKAHNRK NRERAREIAN LAKQWGLSPS
GIL01         MANKRLKKKL ETKRKKSLLV SEG-YSKKET KKLKGRELET VYKKKAHNRK NRERAREIAN LAKQWGLSPS
GIL16c        MANKRLKKKL ETKRKKSLLV SEG-YSKKET KKLKGRELET VYKKKAHNRK NRERAREIAN LARQWGLSPS
AP50          MANKRQRKKI VKKKQESFLS SVG-YSKKQM KTISTTDRAK VVKKETYKKK KRDKYHQARS MGFGS-KEAN
Sato          MAKKRIKKKL EKKKKISLLL SDSSVSKKET KRLKGRELDV VYKQVNQRVK NRERARAISA EAKRWGLSPT
Sole          MAKKRIKKKL EKKKKISLLL SDSSVSKKET KRLKGRELDV VYKQVNQRVK NRERARAISA EAKRWGLSPT

              ....|....| ....|....| ....|....| ....|....| ....|....| ....|....| ....|....|
                     80         90        100        110        120        130        140
Bam35c        KYNSWKKLLP EIERIKKEQD RE-------- -------APF LLIYYQDFTG ETDSKFIYDF KKRNNTRSRS
GIL01         KYNSWKKLLP EIERIKKEQD RE-------- -------APF LLIYYQDFTG ETDSKFIYDF KKRNNTRSRS
GIL16c        KFNSWKKLLP EIERIKKEQD RE-------- -------APF LVIYYQDFTG ETDSKFIYDF KKRTNTRSRS
AP50          KMSSWSDSR- -FIKYIEEFN ---------- -------SYY MIVMYKDVTE ETDSEALHMI KNHTKRRSTS
Sato          KFNSWKKLLP EIERRKKEIA KEEKREEQRR KRAERNKGKA LYVFWTDTQG HSLEEWDRQR DQVEHIYSVH
Sole          KFNSWKKLLP EIERRKKEIA REEKREEQRR KRAERNKGKA LYVFWTDTQG HSLEEWDRQR DQVEHIYSVH

              ....|....| ....|....| ....|....| ....|....| ....|....| *...|....| ....|....|
                    150        160        170        180        190        200        210
Bam35c        QITESIIGWL QNAHNKLFLG R---VAIRIV PKRDVSKTNT LWRNHGYVKI YEGQGKELSK LLTAIETIMV
GIL01         QITESIIGWL QNAHNKLFLG R---VAIRIV PKRDVSKTNT LWRNHGYVKI YEGQGKELSK LLTAIETIMV
GIL16c        QITRSIIGWL QNAQNKLFLG R---VAMRIV PKRDVSKTNT LWKNHGYVKI YEGQGKELTK LLTAIETIMV
AP50          NLLRSIKGWL SVDKNQGYIG G---YEIQVG KKDEIDFHLY AYKQRKYLQA YRGQGLQLKP LLNLLENMMV
Sato          GEEGLRSHIK STIHDRLGIP TGACDAPQIV DDKSQIELTN YYYADGWREV YSGKCRYWLP ILKLIATMMT
Sole          GEEGLRSHIK STIHDRLGIP TGACDAPQIV DDKSQIELTN YYYADGWREV YSGKCRYWLP ILKLIATMMT

              ..*.|....| ....|....| ....|....| ....|....| ....|....| ....
                    220        230        240        250        260
Bam35c        GVYDVKERDK YLKELVAKLR SLPYEKAKKN AKEIQKIYDT KSYKKESWDN DDYY
GIL01         GVYDVKERDK YLKELVAKLR SLPYEKAKKN AKEIQKIYDT KSYKKESWDN DDYY
GIL16c        GVYDVKDRDK YLKQLLNNLR SLPYKQAHRN ANEIQKIYDT KSYTKESWDN DEYY
AP50          LLYMVEAKDQ FVEDLCTNLR KLPYEQAHIN ANYIEEEFIT DRSDLHF--- ----
Sato          ALYKPEDKLQ FVLDLSAEVD YFSNEFAERI HGLIY----- ---------- ----
Sole          ALYKPEDKLQ FVLDLAAEVD YFSNEFAERI HGLIY----- ---------- ----
```

**Figure S4.** Multiple sequence alignment of betatectiviruses terminal proteins (TPs). Sequences were aligned using CLUSTAL W. Asterisks highlight Bam35 TP residues Y172 (in blue) and Y194 (in red) which are conserved among analyzed betatectiviruses, including Sato and Sole. For phage Bam35, conserved residues Y172 and Y194 have been shown to be the priming residue for DNA replication and be involved in the interaction with the DNA polymerase, respectively [3]. The ruler represents amino acids in the multiple sequence alignment. GenBank accession numbers for TPs used in the multiple sequence alignment are: Bam35c, NP_943750; GIL01, CAD59945; GIL16, YP_224102; AP50, YP_002302516; Sato, QWE49625; Sole, QWE49656.

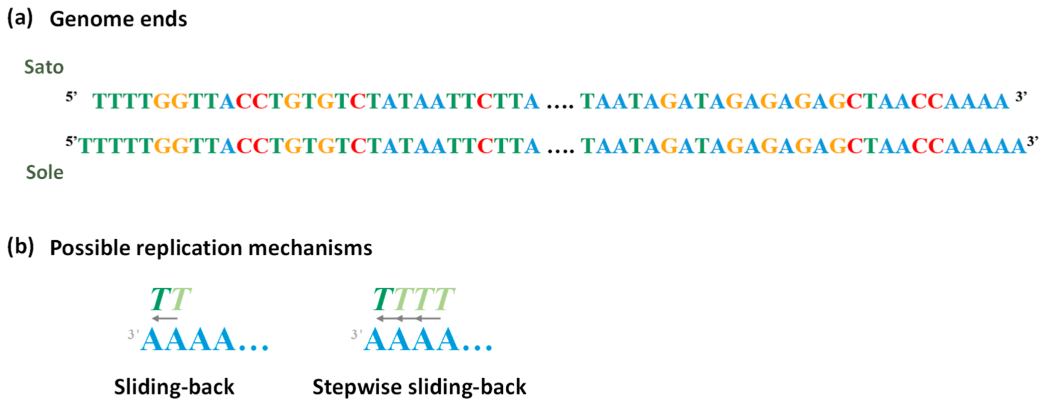


**Figure S5. (a)** Genome ends sequences of betatectiviruses Sato and Sole and **(b)** schematic representation of the possible early steps involved in the protein-primed replication of these phages (adapted from [3]).

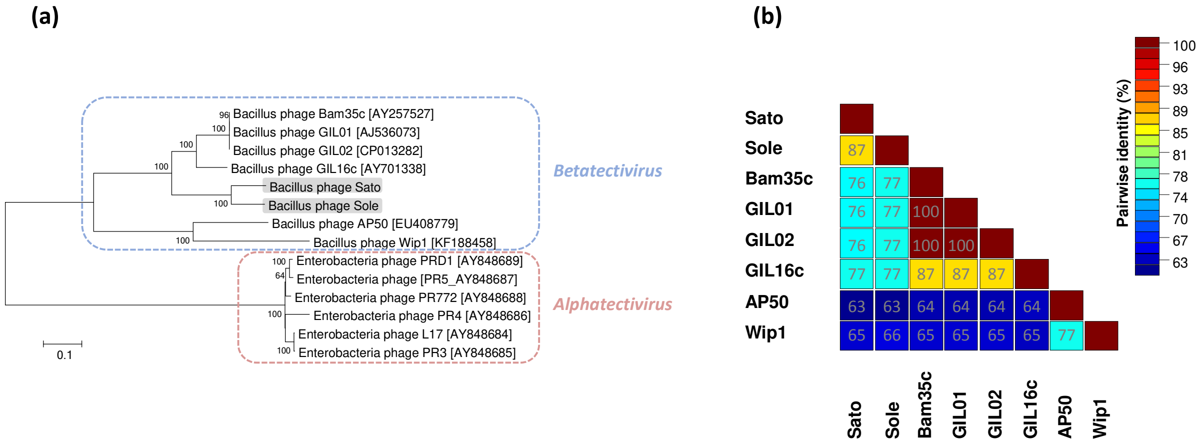


**Figure S6.** Phylogenetic relationships of phages Sato and Sole with other alphatectiviruses and betatectiviruses. **a)** Maximum likelihood tree showing the genetic relationships among the complete sequenced tectiviruses belonging to the genera *Alphatectivirus* and *Betatectivirus*. The general time-reversible plus gamma model of nucleotide substitution was used to build the phylogenetic tree based on complete nucleotide sequences. Bootstrap values (1,000 iterations) above 60% are indicated for each node. GenBank accession numbers are given in brackets. The rose and blue dotted boxes highlight phages belonging to genera *Alphatectivirus* and *Betatectivirus*, respectively. **b)** Graphical representation of percentage of pairwise nucleotide identity (in grey) among fully-sequenced *Betatectivirus*. The color scale bar indicates the percentage of nucleotide identity.

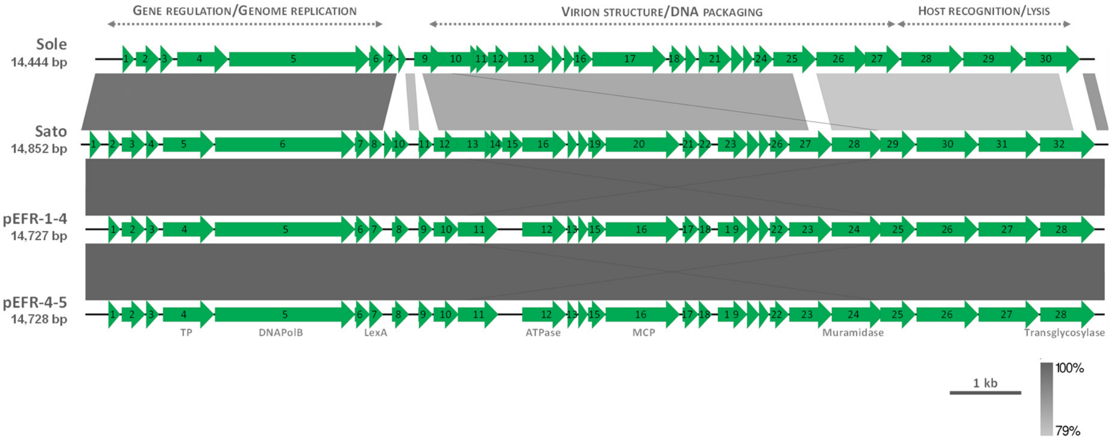


**Figure S7.** Genome comparisons of phages Sato and Sole with *B. cereus s.l.* plasmids pEFR-1-4 and pEFR-4-5. Predicted genes and direction of transcription are represented as green block arrows. CDSs numbers are indicated inside the block arrows. Conserved regions are grey-shaded, with the color intensity indicating the percentage of nucleotide identity from pairwise BLASTn. Scale and percentage of nucleotide identity are indicated at the bottom-right. Genome lengths in base pairs (bp) are indicated for each molecule. GenBank accession numbers are listed in Table 3.

## References

1. Jalasvuori, M.; Palmu, S.; Gillis, A.; Kokko, H.; Mahillon, J.; Bamford, J.K.H.; Fornelos, N. Identification of five novel tectiviruses in *Bacillus* strains: Analysis of a highly variable region generating genetic diversity. *Res. Microbiol.* **2013**, *164*, 118-126.
2. Kelley, L.A.; Mezulis, S.; Yates, C.M.; Wass, M.N.; Sternberg, M.J.E. The Phyre2 web portal for protein modeling, prediction and analysis. *Nat. Protoc.* **2015**, *10*, 845-858.
3. Berjón-Otero, M.; Villar, L.; Salas, M.; Redrejo-Rodríguez, M. Disclosing early steps of protein-primed genome replication of the gram-positive tectivirus Bam35. *Nucleic Acids Res.* **2016**, *44*, 9733-9744.
